# Enhancing Stability and Investigating Target Attainment of Benzylpenicillin in Outpatient Parenteral Antimicrobial Therapy: Insights from In Vitro and In Vivo Evaluations

**DOI:** 10.3390/antibiotics13100970

**Published:** 2024-10-14

**Authors:** Katharina M. Rentsch, Nina Khanna, Delia Halbeisen, Michael Osthoff

**Affiliations:** 1 Department of Laboratory Medicine, University Hospital Basel, 4031 Basel, Switzerland; katharina.rentsch@usb.ch; 2 Division of Infectious Diseases and Hospital Epidemiology, University Hospital Basel, 4031 Basel, Switzerland; 3 Hospital Pharmacy, University Hospital Basel, 4031 Basel, Switzerland; 4 Division of Internal Medicine, University Hospital Basel, 4031 Basel, Switzerland; 5 Department of Clinical Research, University of Basel, 4031 Basel, Switzerland; 6 Department of Internal Medicine, Cantonal Hospital Winterthur, 8400 Winterthur, Switzerland

**Keywords:** benzylpenicillin, therapeutic drug monitoring, stability, degradation, OPAT

## Abstract

**Background/Objective**: Narrow-spectrum beta-lactam antibiotics such as benzylpenicillin and flucloxacillin are increasingly used in outpatient parenteral antimicrobial therapy (OPAT) programs to mitigate the adverse effects associated with broad-spectrum antibiotics. These beta-lactams require continuous administration via portable infusion devices during OPAT. However, the use of benzylpenicillin in OPAT requires special consideration because of its limited stability at elevated temperatures. **Methods**: We tested the benzylpenicillin stability, pH, and degradation of products in elastomeric pumps at different concentrations in saline and in buffered solution containing sodium citrate during a prolonged storage and at high temperatures (seven days at 2–8 °C followed by 24 h at 37 °C). Additionally, drug concentrations during intermittent bolus infusion and during OPAT were determined in five patients. The concentrations and degradation products of benzylpenicillin were measured using liquid chromatography mass spectrometry (LC-MS/MS). **Results**: Unbuffered benzylpenicillin solutions that were already degraded during refrigerator storage and analyte concentration were not measurable after 8 days. The stability of the buffered solutions was acceptable at all three of the tested concentrations (97.6 ± 1.3%, 96.3 ± 0.8%, and 94.9 ± 1.1% for 10 Mio IU, 20 Mio IU, and 40 Mio IU of benzylpenicillin). The stability was influenced by benzylpenicillin concentration, and several breakdown products were identified. Benzylpenicillin concentrations were measured in five patients during OPAT and ranged from 7.2 to 60 mg/L. **Conclusions**: Benzylpenicillin buffered with sodium citrate is a safe and convenient option for use in continuous infusions during OPAT and should be favored over broad-spectrum antibiotics. Therapeutic drug monitoring data indicate sufficient to high plasma levels when patients received benzylpenicillin as continuous infusions.

## 1. Introduction

Intravenous antimicrobial therapy prolongs hospital stays unnecessarily in up to 5% of hospitalized patients [1]. A well-accepted alternative to inpatient care is Outpatient Parenteral Antimicrobial Therapy (OPAT). OPAT refers to the administration of parenteral antimicrobials in an outpatient setting with the explicit aim of facilitating early discharge or avoiding admission, enhancing patient welfare and using hospital resources cost-effectively. Common diseases cared for in an OPAT program include urogenital infections, cellulitis, bone and joint infections, and infective endocarditis. A team approach is paramount for a safe practice of OPAT including careful patient selection, antimicrobial management, and the monitoring of patients and outcomes [2].

The administration of beta-lactam (BA) antibiotics using elastomeric pumps has been associated with good clinical outcome [3]. However, a stability and safety assessment is required for each BA drug. The chemical stability of an active pharmaceutical ingredient is the major limitation for its use in elastomeric pumps, as BAs are easily degraded in watery solutions—this can be as pronounced as almost 100% degradation in solution after 24 h at elevated room temperatures. This has been shown for benzylpenicillin [4]. Continuously administered antibiotics may be exposed to temperatures above 30 °C during OPAT, in particular, when elastomeric pumps are carried close to the body or exposed to the sun [5,6]. Elastomeric pumps release intravenous drug solutions at an almost steady rate. For several antibiotics, it has been shown that drug concentration levels are comparable to those achieved using continuous infusion by standard infusion devices in the hospital setting [3]. However, such evidence is still lacking for benzylpenicillin.

Previously, several groups had reported on the stability of benzylpenicillin at elevated room temperatures, but only one investigated the full time span (8 days) and the full temperature range (up to 37 °C) that might be encountered during OPAT [7,8]. Accordingly, benzylpenicillin was shown not to be sufficiently stable for continuous administration in OPAT programs when applied in sodium chloride 0.9%, and shows a quick chemical degradation [5]. However, the stability of benzylpenicillin may be improved when it is dissolved in citrate-buffered normal saline instead of pure water [4].

In the present study, we investigated the stability of benzylpenicillin at 37 °C in currently used elastomeric pumps according to the British pharmacopeia limit for injections of the 95–105% concentration of the stated amount [9]. In Switzerland, an acceptance limit of up to 10% is usually tolerated. Moreover, we analyzed the degradation products after storage and gathered the therapeutic drug monitoring (TDM) data of five OPAT patients to explore if the plasma concentrations achieved during administration via elastomeric pumps were within the recommended range.

## 2. Results

### 2.1. Pump Flow

**Figure 1:**
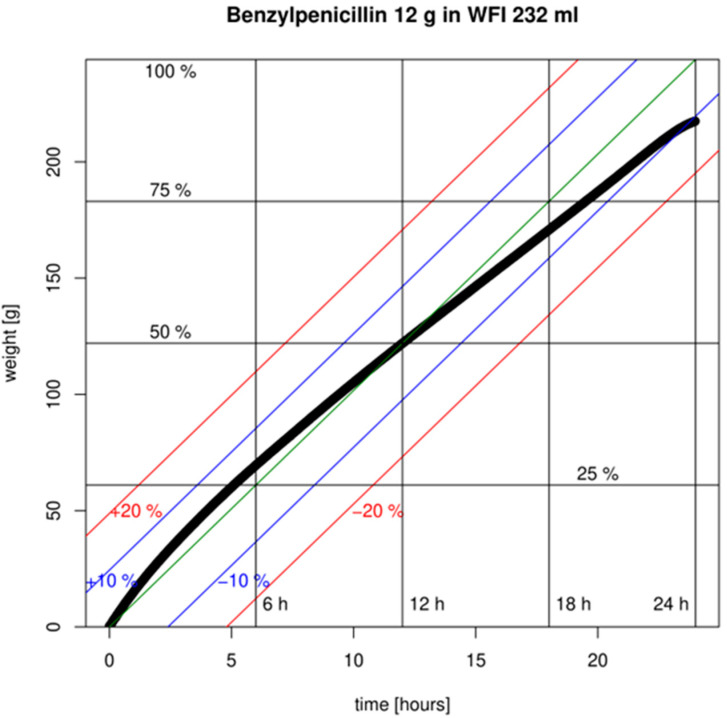
Flow rate (bold black line) of a 20 Mio IU benzylpenicillin pump (standard conc.) prepared with water for injection (WFI). The green line represents the ideal continuous flow rate with deviations of 10 and 20% depicted (blue and red lines, respectively).

The designated pump flow of the Easypump^®^ 270-10-S is expected to be 10 mL/h at 31 °C (flow restrictor temperature). While the measured pump flow exhibited slight time dependence and experienced a gradual decrease over time, it remained stable overall. The flow rate varied by no more than 11% from the target of 10 mL/h (Figure 1).

### 2.2. Stability Analyses of Benzylpenicillin

The validated cold room was set to 2–8 °C and the mean temperature (±standard deviation) over the storage period of 7 days was 4.5 ± 0.9 °C, never exceeding 6.9 °C. The incubator was set to 37 °C but was only able to hold the temperature at a mean of 36.3 ± 2.0 °C due to door handling during sample retrieval.

**Figure 2:**
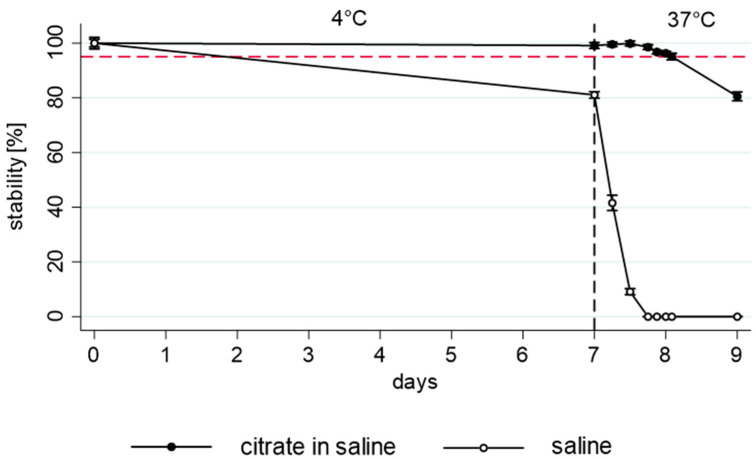
Comparison between buffered and unbuffered 20 Mio IU benzylpenicillin solutions showing the means and standard deviations of the three pumps. The dashed red line represents the 95% stability limit.

**Figure 3:**
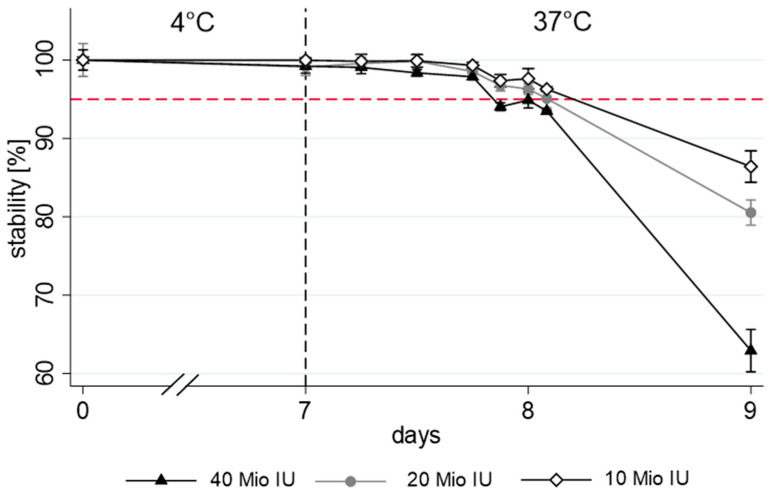
Comparison of three concentrations of benzylpenicillin in citrate buffer showing the means and standard deviations of the three pumps. For easier readability, the 7-day refrigerator period is shown stinted. The dashed red line represents the 95% stability limit.

**Figure 4:**
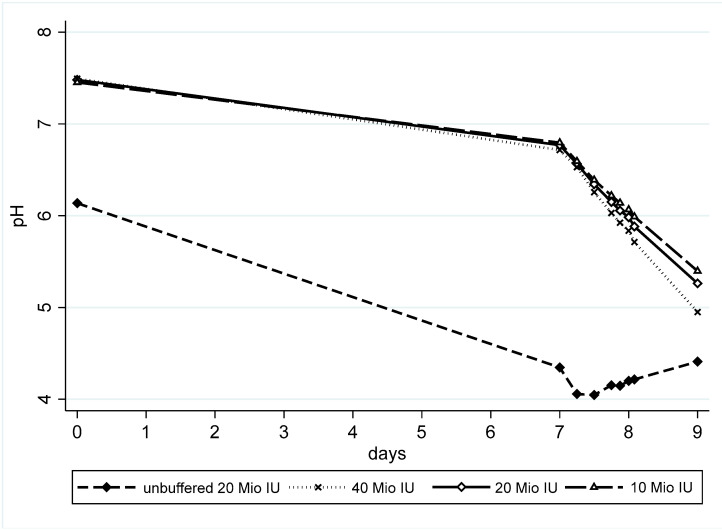
Course of pH in both buffered and unbuffered pumps over time. Since the standard deviations were always below 0.05 pH units, they are not depicted to increase the readability of this Figure.

The results of the stability measurements (quantity of remaining benzylpenicillin and pH) are shown in Figure 2, Figure 3 and Figure 4. The unbuffered benzylpenicillin solutions that were already degraded during refrigerator storage (mean recovery of 81%) had no measurable analyte concentration left after 8 days (Figure 2). Stability was acceptable in buffered solutions at all three tested concentrations. At 10 Mio IU, 20 Mio IU, and 40 Mio IU, 97.6 ± 1.3%, 96.3 ± 0.8%, and 94.9 ± 1.1% of the initial concentration were still available after 8 days, respectively (Figure 3). 95% stability was not achieved only in the highest concentrated infusion during all measurements with a degradation of 6.5% documented after 22 h at 37 °C. When prolonging the 37 °C storage interval to 48 h, significant differences were observed according to the used concentrations (86% ± 2.0, 81% ± 2.7, and 63% ± 1.6 stability for a concentration of 10 Mio IU, 20 Mio IU, and 40 Mio IU, respectively).

In the unbuffered pumps, the mean pH after the preparation of the three pumps was 6.2 and had already dropped during the storage in the refrigerator, reaching a minimal pH of 4.0 (Figure 4). In the buffered pumps, the mean starting pH of all the concentrations was 7.5 and the decrease was much slower.

### 2.3. Degradation of Benzylpenicillin

**Figure 5:**
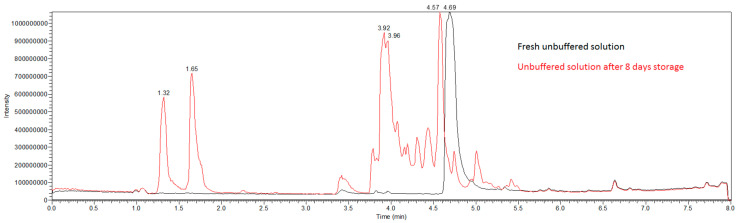
Scans of unbuffered benzylpenicillin solution in elastomeric pumps directly after constitution and after eight days storage: seven days refrigerated at 4 °C and one day at 37 °C.

Several breakdown products in stored benzylpenicillin solutions were identified by qualitative LC MS/MS method. Scans showed the complete disappearance of the benzylpenicillin peak in unbuffered solution after storage, while several new peaks emerged (Figure 5).

**Figure 6:**
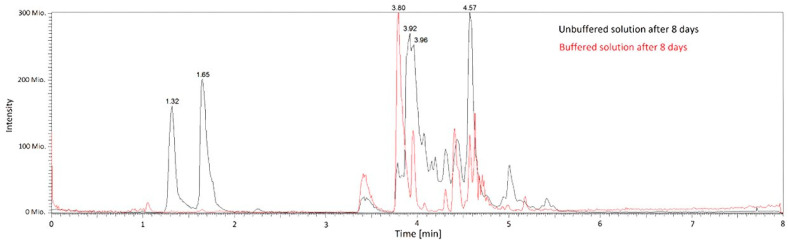
Chromatograms of breakdown products of buffered and unbuffered benzylpenicllin solutions in elastomeric pumps after eight days storage: seven days refrigerated at 4 °C and one day at 37 °C. (Background subtracted chromatograms). Assignment of peaks to different degradation products in the buffered solution is shown in Figure 7.

Subtracting the spectra gained from the fresh solution from a scan of the stored solution yielded higher quality chromatograms with lower background noise (Figure 6). Peaks identified in those spectra were further fragmented using LC-MSMS. Mass spectra were assigned to penilloic acid (retention time 4.57 min), penicillic acid (retention time 3.97 min), penicilloic acid (retention time 3.78 min), isopenillic, and penicillenic acid (retention times 1.32 and 1.65 min).

**Figure 7:**
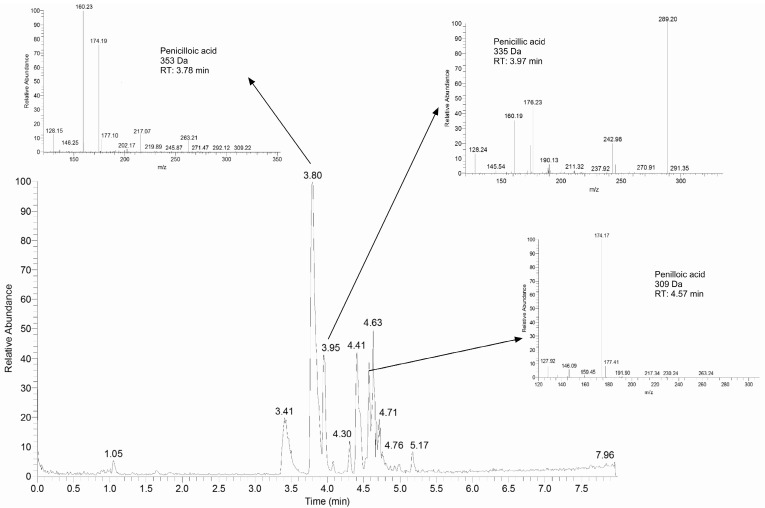
Chromatogram of degradation products in buffered solution. Spectra of some important products are added.

Additionally, different in-source adducts were identified. Details of these experiments may be found in Figure 7 and Table 1.

### 2.4. Therapeutic Drug Measurement of Benzylpenicillin

**Figure 8:**
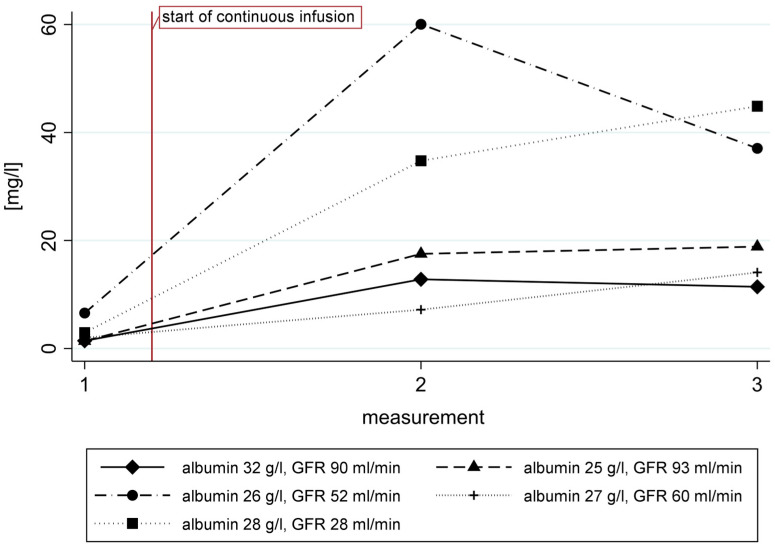
Benzylpenicillin concentrations (mg/L) in five patients receiving benzylpenicillin as intermittent bolus infusion during the first measurement (trough concentration) and subsequently as continuous infusion using an elastomeric pump during OPAT. GFR: estimated glomerular filtration rate in mL/min/1.73 m^2^.

Five patients, who were scheduled to receive benzylpenicillin in the OPAT program for various indications (e.g., infective endocarditis), were included. The standard dosage of benzylpenicillin was 20 Mio IU per day. Only one patient presented with an estimated glomerular filtration rate (eGFR) below 30 mL/min and they received a lower dosage of 15 Mio IU per day. The total daily dosage was identical for all the patients during intermittent bolus administration during admission (4× or 3× 5 Mio IU) as well as during continuous infusion during OPAT. The levels measured after intermittent bolus dosing ranged from 0.5 to 6.6 mg/L. The median time until the first OPAT measurement was 5 days (range of 2 to 10 days), and the second OPAT measurement was taken 14 days (range 11 to 17 days) later. The benzylpenicillin concentrations assessed during OPAT ranged from 7.2 to 60 mg/L (Figure 8) and demonstrated no significant intra-individual variability (two-sided Student’s *t*-test for dependent samples; *p* = 0.85). The creatinine concentration only changed significantly in the patient with the lowest eGFR (increase by 18%); in all the other patients there was no significant increase.

## 3. Discussion

OPAT has been a valuable treatment concept since the 1970s with multiple benefits to a patient, but also to the hospital and societal level. Continuous infusions of antimicrobials that traditionally require multiple daily administrations are the cornerstone of a successful OPAT program. In the present study, the continuous administration of benzylpenicillin in elastomeric pumps was analyzed in depth in vitro and in vivo.

First, the flow rate of drug solution from a contemporary elastomeric pump (Easypump^®^ II LT 270-27-10) was appropriate. Second, the infusions of benzylpenicillin that dissolved in saline (NaCl 0.9%) resulted in insufficient stability for an OPAT program. We decided to use a threshold of 95% as acceptable for the stability of benzylpenicillin due to two different reasons. We aimed to prevent the under-dosing of the antibiotic treatment, as all degradation products do not have an antibiotic activity comparable to benzylpenicillin. Degradation products also may have hazardous effects, which should be minimized, especially in the situation of repeated application of benzylpenicillin in elastomeric pumps. For the saline solution, after 7 days at 2–8 °C, measurements indicated a stability of only 81%, and an additional 90% degradation at 37 °C over the course of 12 h was demonstrated. Buffering with citrate sodium was confirmed to be a reliable way to increase the stability of benzylpenicillin. Specifically at 4 °C, solutions showed no more than 2.2% degradation in any of the nine tested pumps after the first 7 days. At 37 °C, the stability was lower, but still sufficient over the course of 24 h, resulting in a maximal degradation of 6.5% in one of the 40 Mio IU pumps. This demonstrates that benzylpenicillin dissolved in citrate buffered solution enables a safe use in patients for doses up to 20 Mio IU with the more stringent stability threshold of 95% stability. The conditions tested depict a worst-case scenario, in which infusion pumps are stored for a whole week and are subsequently exposed to high temperatures during infusion, when pumps are directly exposed to sunlight during the summer or in areas with elevated average temperatures. Differences in concentration dependent degradation became more pronounced after a prolonged storage at 37 °C for 48 h. The amount of added citrate buffer was proportional to the benzylpenicillin dose. Thus, with lower or higher benzylpenicillin concentrations buffer concentrations were also adapted. The lowest concentrated analyte buffer in the NaCl 0.9% mixture resulted in the lowest degradation rate. Our results are in line with the study of McDougall et al. [4] and Vella-Brincat et al. [5] concerning the stability of benzylpenicillin in elastomeric pumps in unbuffered and buffered solutions. We demonstrate a slightly increased stability after storage at 37 °C for 24 h with 4% (1–6%) degradation in a concentration range of 10–40 Mio IU as compared to 7% (4–9%) for a concentration of 6–24 Mio IU [4]. One explanation for the difference could be that the elastomeric pumps used in the two studies have not been manufactured by the same company. As we demonstrate an increased degradation with increasing concentration, an even higher instability must be expected for a concentration of 40 Mio IU (compared to the highest analyzed concentration of 24 Mio IU in the study of McDougall et al. [4]). The observed concentration-dependent stability of benzylpenicillin contrasts with previous studies, which may be related to the short high-temperature interval chosen in previous studies [4,5]. In the present study, the difference in stability, while already visible after 24 h, became obvious during storage at 37 °C for 48 h.

Previous publications have demonstrated that the stability of benzylpenicillin is influenced by pH, with increased degradation observed under both acidic and alkaline conditions [10]. According to this theory, the pH is expected to decrease to a lesser extent in solutions with lower concentrations. Our pH measurements align with this theory. Starting pH decreased over time in both buffered and unbuffered pumps. Since two major degradation products, penicilloic acid and penillic acid, have two carboxyl groups, while benzylpenicillin has only one, analyte degradation may account for the decreasing pH. The additional carboxyl group provides an additional [H^+^], thereby decreasing pH. When these two products are further degraded, they again lose the second carboxyl group, resulting again in a rise in pH. The pH dropped about 0.7 units during the 7-day refrigerated storage period in buffered solutions. At elevated temperatures, we observed an almost perfect linear decline in pH for the buffered solutions, correlating with the roughly exponential decrease in benzylpenicillin content. For the unbuffered pumps the decline was more pronounced in the cold phase and terminated in a more complex pattern in the warm phase. This is probably related to the complex build-up and breakdown of different degradations products and their varying pKa_S_ at this advanced degradation state. We support this with our data by observing the exponential change in [H^+^] ions, indicated by the linear pH decline. If the degradation was not self-accelerating, we would observe an almost linear benzylpenicillin concentration decay and a non-linear pH decay. Interestingly, the spontaneous hydrolysis of benzylpenicillin is prevented by deprotonation of the carboxyl group, which is pH-dependent (increased hydrolysis in acidic media compared to physiological conditions) [11].

The major degradation products we have identified in the buffered solution are penicilloic acid, penicillic acid, and penilloic acid. These compounds are well-known, mainly in the food industry, as they may be present in livestock treated with benzylpenicillin [12]. In mice, they described the potential toxic effects of higher doses of benzylpenicllin degradation products. The same authors studied the toxic effects of penilloic acid [13]. Whereas the allergic reactions of this degradation product have been described before, they demonstrated its cytotoxicity in different cell-culture systems. In another study in mice, penilloic acid has been described to also be the major culprit of the non-allergic hypersensitivity reactions in penicillin treatment [14]. Penicillic acid is well-known as mycotoxin, which has antibiotic activity and is cytotoxic, hepatotoxic, and carcinogenic [15,16].

Third, several breakdown products were identified in the qualitative analyses of the pump solutions, including penilloic acid, penicilloic acid, and penillic acid. Penicillenic acid and isopenillic acid were tentatively identified, but only in unbuffered solutions. Benzylpenicillin and benzylpenicillenic acid have been identified as forming allergy-inducing protein-conjugates, with benzylpenicillenic acid being the more reactive species [17]. Our results are in line with the breakdown processes postulated by several authors, which agree, that both degradation products are formed later on (during the breakdown process) from other breakdown products [18,19]. While liquid chromatography revealed several peaks in the degraded infusion solution, not all of these could be identified. Most of the observed peaks were identified as adducts of benzylpenicillin or one of its breakdown products. None of the other metabolites found in humans such as penamaldic acid, penilloaldehyde, aminopenicillanic acid, penaldic acid, or penicillamine were found in the degraded solution, even after complete degradation, which was reported before [20,21].

Last, we report data from five patients included in the local therapeutic drug monitoring (TDM) program. During intermittent bolus infusion, all the patients presented with low benzylpenicillin trough levels. During continuous infusion, sufficient to high levels of benzylpenicillin were observed in all the patients despite identical total daily dosages. In critically ill patients or for difficult-to-treat infections (such as infective endocarditis or vertebral osteomyelitis) concentrations of BA above the MIC, or even for times above the MIC, should be targeted for the entire dosing interval [22]. The European Committee on Antimicrobial Susceptibility EUCAST defines non-species-specific benzylpenicillin breakpoints at 2 mg/L. However, the breakpoints for most bacteria are lower [23]. The levels measured in our study cohort were therefore appropriate to elevate during OPAT compared to levels slightly too low during intermittent bolus infusions. In addition, interindividual variations in the benzylpenicillin serum concentrations were large, which is a characteristic finding of most BA [24]. Although the therapeutic index of benzylpenicillin is assumed to be wide, adverse events related to excessive concentrations may happen. In particular, benzylpenicillin has a high relative pro-seizure activity [25] that may become evident in the case of renal impairment or concomitant seizure threshold-lowering drugs. Since constantly elevated levels probably increase toxicity, TDM may be performed during continuous infusion to decrease the risk of toxicity or lower daily dosages be administered.

Limitations include the use of only one pump for the assessment of the flow rate. However, our practical experience in the OPAT program supports the pump to be suitable for benzylpenicillin administration. Furthermore, due to the open system used, the solution partly evaporated during measurement. This effect was not compensated for and may lead to a potential underestimation of the flow rate and the total delivered mass. We only tested the stability at 37 °C, but not at 31 °C or 32 °C as suggested by the United Kingdom’s “Guidance on the Pharmaceutical Issues concerning OPAT and other outpatient intravenous therapies” which was issued in 2018 by the NHS Pharmaceutical Quality Assurance Committee for stability testing [26]. Although pump solution temperatures of 32 °C are rarely exceeded, maximum temperatures of up to 36 °C have been reported in patients [5]. The strengths include the larger tested concentration range of 10 Mio IU to 40 Mio IU compared to previous studies, which may reassure clinicians and pharmacists when choosing different daily dosages, and the use of a different elastomeric device. A 240 mL pump containing 47 mg/mL benzylpenicillin corresponds to a standard daily dose of 20 Mio IU, while 10 Mio IU pumps are of interest in pediatric cases or in patients with impaired renal function. Higher daily dosages of 40 Mio IU may be needed in the treatment of endocarditis or meningitis.

## 4. Materials and Methods

### 4.1. Materials

The benzylpenicillin potassium salt (Sigma Aldrich, Buchs, Switzerland) and benzylpenicillin-d5 potassium salt (Toronto Research Chemicals, Toronto, ON, Canada), were both of analytical grade. Penicillin “Grünenthal” 10 Mio IU for infusion (Grünenthal Pharma AG, Mitlödi, Switzerland) containing 10 million international units of benzylpenicillin (i.e., 5.988 g of benzylpenicillin sodium) was used to fill the elastomeric pumps (Easypump^®^ II LT 270-27-S (B. Braun Medical AG, Sempach, Switzerland)). For preparation of the elastomeric pumps, sodium chloride (NaCl) 0.9% for injection (B. Braun Medical AG, Sempach, Switzerland) and in-house pharmacy-produced 40 mg/mL sodium citrate solution (prepared with sodium citrate 3.13% Ph. Eur. (Hänseler AG, Herisau, Switzerland)) where used.

### 4.2. Measurement of Benzylpenicllin

For the quantification of benzylpenicillin, a previously described LC-MS/MS method was adapted [27]. After online extraction using a Thermo (Waltham, MA, USA) TurboFlow MAX column, the analytes were separated on an Acuccore^TM^ XL C18 4 µm column (both Thermo Fisher Scientific, Reinach, Switzerland). Mobile phase A was composed of 10 mM ammonium carbonate in water, adjusted to pH 8 with acetic acid, and mobile phase B was composed of 10 mM ammonium acetate and 0.1% formic acid in methanol/acetonitrile (1/1, *v*/*v*). They were used in gradient mode. The mass spectrometer (TSQ Endura, Thermo Fisher Sientific, Reinach, Switzerland) was operated in positive mode after electron ion spray ionization to increase the accuracy and precision of the deuterated benzylpenicillin that was used as the internal standard for all the measurements. The measured parent masses for benzylpenicillin and benzylpenicillin-d5 were *m*/*z* 335.0 and 340.2, respectively. The lens voltage was kept at 126 V for benzylpenicillin and 139 V for benzylpenicillin-d5. The product ions and their respective collision energies were *m*/*z* 114.1 (31.5 V), 160.1 (10.3 V), and 176.1 (11.5 V) for benzylpenicillin and 114.1 (32.4 V), 160.1 (10.3 V) and 181.1 (13.2 V) for benzylpenicillin-d5, respectively. Patient samples were measured according to the described protocol, while drug infusions were diluted 500 times with water before work up.

### 4.3. Identification of Degradation Products

To identify the degradation products of benzylpenicillin, qualitative measurements of the drug infusions were performed by adapting the HPLC method to exclude the online extraction, as a clean-up of the samples was not necessary. The MS was first used in scan mode, measuring masses from 145 to 1000 *m*/*z* at a scanning speed of 1000 Da/s. In further measurements product scanning mode was employed. Parent masses identified in scan mode were further fragmented using 1.5 mTorr collision gas and collision energy ramped from 15 to 35 V, while the scanning speed was kept at 1000 Da/s. The samples were diluted 1:50 before qualitative measurement. Where available, the breakdown products were identified with the help of previously published analyte spectra [18,20,21].

### 4.4. Study Design

The elastomeric pump flow of a single pump over 24 h was measured at room temperature. The flow restrictor was led through a warm water bath of about 31 °C (mimicking skin temperature) and the output was dripped into a beaker on a balance. The balance readout was taken automatically every 60 s via computer script. The pump was filled with a standard dose (20 Mio IU) of Penicillin “Grünenthal” in water.

For the drug stability assessment and the treatment of patients the pumps where prepared as follows: each vial of Penicillin “Grünenthal” 10 Mio IU was dissolved in 17 mL sodium citrate 40 mg/mL or sodium chloride 0.9%. The appropriate amount of sodium chloride 0.9% required to achieve a total volume of 240 mL was then added to the pump. Finally, the benzylpenicillin solution (two or four vials per pump in either one) was added to the elastomeric pump. The pumps were filled with three different amounts: 10 Mio IU, 20 Mio IU, and 40 Mio IU, corresponding to a concentration of 23, 46 and 93 mg/mL benzylpenicillin. For each concentration, three pumps were prepared. The unbuffered pumps were prepared by dissolving the drug product in 0.9% sodium chloride only. After preparation and before taking the first sample, the pumps dedicated for the stability study were gently shaken to mix the solutions carefully.

The elastomeric pumps were kept in a cold room at 2–8 °C and an incubator at 37 °C. Both were temperature controlled and a ventilator circulated air inside to ensure uniform air distribution. At the allocated time points, samples were drawn and kept frozen at −80 °C until measurement. Samples were drawn immediately after the preparation of the pumps, after 7-day refrigeration (168 h), including a 30 min warm up phase at room temperature, as well as after 6, 12, 18, 21, 24, 27, and 48 h at 37 °C.

At each sampling point three 1.5 mL sampling vials were filled from each pump with about 1 mL solution by opening the valve and inserting the tube in the sampling vials. The first sample was discarded, the second was dedicated as a pH and backup sample, and the third was used for quantitative analytics. At point ‘zero’, following the preparation of the pumps, two additional blank samples and one extra sample for osmolality testing were collected.

The pH values of the samples were measured using a Metrohm 780 pH meter equipped with a microelectrode “Biotrode” (Methrom Schweiz AG, Zofingen, Switzerland).

### 4.5. Patients

The patients receiving benzylpenicillin as intermittent bolus infusion in the hospital and later continuing the treatment using elastomeric devices during OPAT were included, if TDM measurements had been performed. The trough level is usually measured during the intermittent bolus administration of benzylpenicillin. The second measurement is then performed after at least one 24 h continuous infusion using elastomeric pumps during OPAT treatment and the third measurement is conducted at least five days after the second. Benzylpenicillin measurements were part of the routine TDM program at our hospital (including the measurement of BA antibiotic concentration administered as continuous infusions). Both the blood samples during OPAT were collected during the patient’s weekly visits at the OPAT unit. Internal guidelines recommended dose adjustments to achieve a target of 100% *t*T_>2-4xECOFF_ or if the MIC of the pathogen was known 100% *t*T > 2-4xMIC. Although no formal toxicity threshold was set, benzylpenicillin concentrations above 40 mg/L (i.e., 20xECOFF) usually triggered a dose reduction. Renal function was estimated using creatinine measurements from patient data and eGFR using the Chronic Kidney Disease Epidemiology Collaboration study equation. Mean values were calculated from blood samples taken on all the study days, and standard deviations (SD) were calculated.

### 4.6. Ethics

Blood samples were taken as part of a routine assessment of the local OPAT program. All the patients included had consented to the use of their routinely collected and available clinical and laboratory data (informed consent of the University Hospital Basel), which is the requirement of the local Ethics Committee of Northwest and Central Switzerland for the use of the data in reports and publications. Patients were included over a one-year period according to the availability of the informed consent.

## 5. Conclusions

In conclusion, benzylpenicillin buffered with sodium citrate is a safe and convenient option for use in continuous infusions during OPAT and should be favored compared to broad-spectrum antibiotics whenever possible. TDM data showed sufficient to high plasma levels when patients received benzylpenicillin as continuous infusions during OPAT.

## Figures and Tables

**Table 1:** Identified degradation products and their adducts. The retentions times, parent and product masses, and their relative intensity are shown. Most of the degradation products are known to be allergenic and/or toxic in higher concentrations.

Analyte	Retention Time [min]	Parent Mass [*m*/*z*]	Product Masses [*m*/*z*] (Relative Intensity)
Benzylpenicillin (M)	4.75	335	176.2 (100), 160.2 (90)
Penilloic acid	4.57	309	174.4 (100), 177.4 (10), 127.9 (10)
Penillic acid	3.95	335	289.1 (100), 176.1 (50), 160.1 (35), 243.1 (30), 174.1 (15), 128.1 (15)
Penicilloic acid	3.41 + 3.78	353	160.2 (100), 174.2 (80), 217.2 (15), 128.2 (15), 177.1 (10)
Isopenillic acid/Penicillenic acid	1.32/1.65	335	159.1 (100), 185.1 (65), 203.1 (30), 283.2 (25), 289.2 (20)
Predicted Adducts			
[2 Penillic acid + Na]+	4.15 + 4.41	691	357.2 (100)
[M + CH_3_OH + H]+	4.63	367	335.1 (100), 160.1 (10), 176.1 (10)
[2 Penillic acid]+	4.20	669	335.2 (100), 289.2 (10), 160.1 (10), 176.1 (10)
[2 M]+	5.20	669	335.2 (100), 217.0 (35), 317.3 (30), 176.1 (10), 160 (10)
[2 Penilloic acid]+	4.30	616.4	309.2 (100), 160.1 (15), 263.2 (15)

## Data Availability

The datasets used and/or analyzed in the current study are available from the corresponding author upon reasonable request.
